# 

**Published:** 1921-05-07

**Authors:** 


### THE COLLEGE OF NURSING

(Limited by Guarantee.)

### What the Local Centres are Doing.

### CARDIFF CENTRE.

(Hon. Secretary, Miss M. E. Hitch, Anthony House, 30 Newport Road.)

Thursday, May 12, 8 p.m., in the Anatomy Lecture Theatre, Old College Buildings, Newport Road, Professor D. Hepburn, Professor of Anatomy at the University, Cardiff, on "The Lymphatic System," illustrated by lantern slides. Admission free to members of Centre; non-members, 1s.

### BIRMINGHAM THREE COUNTIES CENTRE.

(Hon. Secretary, Mrs. Glegg, 84 Hagley Road.)

Thursday, May 12, at 5.30 p.m., in the Lecture Theatre of the General Hospital, Birmingham (by kind permission of the Governors), Dr. T. C. Graves, M.D., F.R.C.S., Medical Superintendent, Rubery Hill Mental Hospital, on "The Position of a Knowledge of Mental Diseases in the General Nursing Council Curriculum." Admission: Members, free; non-members, 1s.

### Attempt to Boycott the College of Nursing.

There was a curious discussion at a recent meeting of the Swansea Workhouse Visiting Committee on a letter from the superintendent nurse, Miss M. B. Williams, who intimated her appointment as Secretary of the Swansea Centre of the College of Nursing. Miss Williams said the work attaching to the office would be performed in her own spare time, but she had put the matter before the Committee out of courtesy. Several members objected to Miss Williams accepting the post on the ground that the College was really a trade union. Eventually, however, no action was taken in the matter, and Miss Williams is free to do as she pleases.

### Lectures to Creche Workers.

Under the auspices of the National Association for the Prevention of Infant Mortality and the Welfare of Infancy, and the National Society of Day Nurseries, the course of advanced lectures on "Infant Care," especially intended for crèche nurses and probationers, will be held at the Essex Hall, Essex Street, Strand, W.C. 2, on Thursdays, from 7.30 to 8.30 p.m., from May 5 to July 21. This course is in preparation for the crèche nurses' certificate. The course is equally suitable for voluntary workers in day nurseries and others, who are invited to attend the lectures, the fee for which is 10s. Tickets can be obtained either from Miss Halford, Secretary, National Association for the Prevention of Infant Mortality, 4 and 5 Tavistock Square, London, W.C. 1, or Miss Maddock, Secretary of the National Society of Day Nurseries, 20 Berkeley Street, S.W. 1.

### Notification of Chicken-Pox.

The Scottish Board of Health have issued Regulations extending the period in which chicken-pox is compulsorily notifiable until October 1921.

### Hospitals and Approved Societies.

With reference to an inquiry issued recently by the British Hospitals Association as to the view taken by individual hospitals on the question of payment for insured patients, we are requested to state that it is hoped that an answer may be sent to the Association at as early a date as possible, in order that the negotiations between the Association and the Approved Societies may continue without undue delay.

### New Hospital for Neurasthenics.

THE KING AND QUEEN have graciously consented to become patrons of a new hospital or sanatorium for the treatment of functional nervous disorders, which through the generosity of Sir Ernest Cassel is to be founded at Penshurst, Kent, where a large mansion has been purchased and adapted to accommodate sixty patients. To this object Sir Ernest Cassel has devoted the sum of £225,000. It will be remembered that on several occasions previously his generosity has been directed towards the cause of the sick and suffering, notably by the gift of £50,000 in 1911, and a like amount in 1915, to King Edward's Fund for London, in memory of his daughter, the late Mrs. Ashley. It was to his munificence, too, that the establishment of the King Edward VII. Sanatorium at Midhurst was due.

Sir Ernest is the Chairman of the General Committee, and the Medical Committee comprises the following: Miss Aldrich-Blake, M.S., Dr. Farquhar Buzzard, Sir Maurice Craig, Lord Dawson, Professor J. S. Haldane, Dr. Henry Head, Dr. A. F. Hurst, and Sir Frederick Treves. Dr. T. A. Ross, who has a wide experience of diseases of the nervous system, has been appointed medical director.

The upkeep of the institution is largely provided for by the endowment, but a charge is being made to each patient towards the cost of maintenance. The hospital will be ready for patients on May 23, and applications should be made to the Medical Director, Cassel Hospital, Swaylands, Penshurst, Kent.

### "Truth About Venereal Disease."

WITH reference to this book, which was reviewed in these pages on March 26, the publishers, G. P. Putnam's Sons, Ltd., inform us that proof-sheets were submitted, among others, to Professor W. M. Bayliss, Sir James Crichton-Browne, Dr. Jane Hawthorne, Sir W. Arbuthnot Lane, and Sir John MacAlister, and that all of the above have read the proof-sheets and given permission to quote their names in approval.

May 7, 1921] [Price Twopence

### The Hospital

**RATES OF SUBSCRIPTION** (post free, payable in advance).

Three months, 3/3; six months, 6/6; twelve months, 13/-. (Should the cost of printing and paper as well as increased postage, necessitate alteration of these rates, the period paid for will be reduced proportionately on unexpired subscriptions.) **THE HOSPITAL "Index" to Volume LXIX., October 1920 to March 192 , is now ready, price 1/1 per copy, post free.** Address The Manager, THE HOSPITAL, The Hospital Building, 28/29 Southampton Street, Strand, London, W.C. 2.

